# Enhancing Cooperation in 5–6-Year-Old Rural Chinese Children through Cooperative Constructive Play Based on Anji Play: A Quasi-Experimental Study

**DOI:** 10.3390/bs14070533

**Published:** 2024-06-25

**Authors:** Xinya Lin, Yunpeng Wu, Jianfen Wu, Liping Qin

**Affiliations:** 1 Zhejiang Philosophy and Social Science Laboratory for Research in Early Development and Childcare, Hangzhou Normal University, Hangzhou 310030, China; 2022112004250@stu.hznu.edu.cn; 2 School of Teacher Education, Dezhou University, Dezhou 253023, China; lp_qin@hotmail.com

**Keywords:** cooperation, constructive play, Anji Play, child development, educational intervention

## Abstract

Cooperation plays a crucial role in children’s social development and adaptation. This study designed a cooperative constructive play (CCP) intervention based on the Anji Play teaching model and evaluated its effectiveness in a quasi-experimental design involving 60 rural Chinese children aged 5–6 years. Participants were divided into an experimental group, which engaged in 12 weeks of CCP, and a control group, which continued with regular teaching activities. The cooperation data were collected through a truck racing task using pre-, mid-, post-, and follow-up tests, while the cooperation levels and strategies were evaluated by video observations of role-playing games before and after the tests. The results indicated significant improvements in cooperation scores in both the truck racing task and role-playing games in the experimental group compared to the baseline, with no similar enhancements observed in the control group. Furthermore, cooperation scores in the follow-up were higher than pre- and post-test scores, demonstrating the CCP’s effectiveness in fostering child cooperation, transferability to other contexts, and sustainability. These findings suggest that CCP intervention based on Anji Play can significantly enhance cooperation in children, offering a valuable tool for educational practices.

## 1. Introduction

Cooperation is a fundamental aspect of human social interaction that begins to emerge in early childhood. It involves the ability to work together towards a common goal, which is a critical skill for children as they develop socially and cognitively. Anji Play, originating from Anji County, Zhejiang Province, China, has expanded its educational philosophy widely within China and internationally into Europe, the Americas, and Africa. Anji Play is characterized by its unique outdoor activities, which provide ample time and space for interpersonal interactions among children, thereby facilitating the emergence of cooperative behaviors. However, empirical studies on cooperative constructive play under the Anji Play teaching model, especially in rural areas of China, are lacking. Therefore, there is a need to design a cooperative training program in China to assess its effectiveness in enhancing children’s cooperation.

### 1.1. The Definition and Importance of Cooperation

Cooperation is a fundamental aspect of early development, where children engage in understanding a common goal and collaborating to achieve it [1]. Even in children as young as 3.5 years old, early cooperative behaviors, such as sharing and reciprocity, are already evident, indicating that the foundations of cooperation are established early in life [2]. As children grow older, the complexity and frequency of cooperative interactions increase, reflecting their developmental progress.

Collaboration not only supports but also significantly enhances cognitive development among children aged 4 to 7 years. It provides immediate benefits for complex cognitive tasks that involve visual perception, problem solving, and rule-based thinking [3]. Moreover, the act of collaboration itself can heighten motivation among preschoolers, especially for tasks that pose a challenge, suggesting that the nature of collaborative activities is intrinsically motivating for children [4]. The ability to engage in successful peer cooperation is not static; it grows with age and is influenced by both inherent temperamental traits and the quality of prior peer interactions [5]. This progression underscores the evolving nature of cooperative skills, as children not only learn from, but also build upon, their previous experiences in social contexts. 

### 1.2. Cooperation Interventions in Early Childhood

Research consistently demonstrates that well-designed educational interventions can significantly enhance cooperation among children. Cooperative play is particularly effective in improving interaction and cooperation through structured activities like cooperative math games, which engage children and build foundational social skills [6]. In studies involving thematic joint block-building games for children aged 4–5, it has been observed that children in the experimental groups display higher cooperation levels than those in the control groups [7]. Furthermore, just four weeks of cooperative play training can substantially enhance cooperative behaviors in preschoolers, highlighting the effectiveness of immersive, interactive learning experiences [8]. 

Beyond traditional games, other methods also foster cooperation. For instance, collective singing activities have been shown to cultivate cooperation more effectively than competitive games, demonstrating that cooperation can be nurtured through various group activities [9]. Similarly, synchronized movement activities that require children to move in unison enhance cooperation, evidenced by quicker completion of joint tasks and improved intentional communication [10]. 

Moreover, child-directed play contexts, where children have greater control over task goals and interactions, promote not only higher levels of cooperation but also more effective learning and performance [11]. This is supported by the implementation of arts and crafts activities, in which group work is used to improve collaboration skills from an early age [12]. 

These findings collectively underscore the importance of incorporating diverse and engaging cooperative activities within educational settings to foster essential social and cognitive skills among children, paving the way for enhanced interpersonal relationships and academic success as they grow. 

### 1.3. The Advantages of Constructive Play

Constructive play involving children manipulating building blocks not only facilitates sensory experiences and psychological satisfaction, but also promotes essential social interaction and construction skills [13]. This type of play is highly favored among children, with more than half preferring constructive activities over other types of play due to their engaging and interactive nature [14]. In educational settings, the area designated for constructive play acts as a vibrant social environment in which children naturally communicate and collaborate, thereby fostering an atmosphere conducive to social learning [15]. Using building blocks, children develop a range of skills that are critical not only to their immediate play activities but also to their overall cognitive and social development. These skills include cooperation, role-playing, categorization, grouping, comparison, arrangement, and decision-making [16]. 

Moreover, participation in cooperative constructive play serves as a collective endeavor that not only engages children, but also helps dismantle psychological barriers between individual players and the group. This dynamic facilitates the frequent emergence of cooperative behavior, a crucial component in the development of teamwork and empathy [17]. Educators are encouraged to provide ample time for these activities and to supply diverse, open-ended materials that spur creativity and innovation. Such resources allow children the flexibility to explore various constructions and solutions in a less structured environment, thereby enhancing their ability to think critically and solve problems creatively [18]. 

Ultimately, constructive play is celebrated for its dual role in offering joy and learning opportunities. It provides a foundation upon which children can build complex social relationships and cognitive abilities, making it an invaluable component of early childhood education. Through the strategic use of constructive play, educators can significantly boost children’s cooperative capabilities and prepare them for future academic and social success. 

### 1.4. Anji Play

Anji Play, developed by Cheng Xueqin in Anji County, China, is an early childhood education curriculum rooted in the philosophy of “True Play”. This model posits play as a fundamental activity for children, aiming to “light up children’s lives” by empowering them to take control of their play experiences [19]. Anji Play encourages children to explore, innovate, and express themselves freely, fostering deep engagement with their learning environment.

Anji Play emphasizes children’s self-directed play, exploration, and discovery using simple natural materials in minimally structured environments. The pedagogy is child-centric, focusing on self-determined play, during which children have the autonomy to choose what, where, and with whom to play. Teachers act primarily as observers and facilitators, intervening minimally to support the children’s initiatives [20,21]. The environments in Anji Play are specially designed indoor and outdoor play spaces equipped with open-ended, natural materials, such as ropes, bamboo ladders, clay pots, and everyday objects like tires and barrels. These materials encourage creative exploration and construction, promoting a hands-on learning experience. Technology integration is also a key element, with teachers documenting children’s play through photos and videos. These recordings are reviewed with the children to facilitate reflection and discussion, enhancing their understanding and engagement.

Anji Play represents a significant shift from traditional early childhood education methods, which are often teacher-directed and structured. Instead, Anji Play adopts a play-based exploratory model that values children’s autonomy and self-determination. Unlike traditional classrooms with prescribed learning areas, Anji Play environments are open and flexible, designed to stimulate natural curiosity and interaction.

Teachers in Anji Play observe and document children’s play rather than direct it. They use these observations to facilitate reflective discussions with the children, helping them build knowledge and develop critical thinking skills. While traditional methods often separate play from learning, Anji Play integrates them, viewing self-directed play as the primary means for children’s learning, growth, and development. Anji Play also encourages children to take appropriate physical, emotional, and intellectual risks, contrasting with traditional methods that often prioritize safety over exploration [22].

A core component of Anji Play is reflection, during which children discuss their play experiences, fostering deeper understanding and learning, unlike traditional teacher-led instruction. This holistic model ensures that children are respected as active participants in their learning journey and supports their right to true play, self-expression, and discovery.

### 1.5. Cooperative Constructive Play Based on Anji Play

The Anji Play curriculum is meticulously structured into three primary stages: preparation, free play, and interpretation. These stages are designed to guide children through a process of self-directed exploration, followed by reflective learning. The designated play areas, such as construction zones, climbing areas, and sand–water areas, are crafted to facilitate diverse interactions with materials and peers, which significantly enhances both motor skills and social capabilities [23].

Anji Play advocates natural ecological environments, the use of large, low-structure materials, and high-quality teachers. Currently, many kindergartens in China have implemented Anji Play and have been designated Anji Play practice kindergartens. However, it is challenging for regular rural kindergartens to implement Anji Play for three main reasons.

First, in terms of the environment, Anji Play practice kindergartens create natural play areas with connected indoor and outdoor spaces. These environments include challenging elements, such as the various heights of slopes, caves, and ravines, where children can climb, roll, and slide. They also have expansive outdoor construction areas where children can engage in play. In contrast, regular rural kindergartens typically lack natural environments and are often limited to rubber playgrounds with separate indoor and outdoor spaces. Play in these settings usually involves small block constructions within the classroom.

Second, regarding materials, Anji Play practice kindergartens provide children with low-structure natural materials, including bamboo ladders, wooden boards, barrels, and tires. Children can freely select these materials, which often require cooperation to transport and use during play. However, regular rural kindergartens mostly use uniformly purchased blocks and other materials. Although some large Anji Play materials have been introduced, they are not utilized effectively.

Third, in terms of teaching staff, teachers at Anji Play practice kindergartens receive professional training that enables them to observe and document children’s activities, intervene minimally, and guide children in adventurous and cooperative play. On the other hand, regular rural kindergartens often have weaker teaching staff and fewer training opportunities. Teachers in these settings tend to exert high control over children’s play and do not encourage adventurous activities.

To address these challenges, we designed a cooperative constructive play program tailored for use in these kindergartens. This program integrates aspects of the Anji Play method with the day-to-day activities of regular kindergartens, ensuring that the ecological essence of Anji Play is maintained in outdoor constructive play while preserving the kindergarten’s routine. Previous research on Anji Play involving rural children designed a metacognitive game-circling curriculum and conducted a three-month intervention with 5–6-year-old children in the experimental group. The findings revealed that this curriculum significantly enhanced the development of metacognitive abilities in young children [24]. Inspired by this study, our research’s activities unfold in three structured stages:

1. Teacher–child dialogic interaction. This forms the core of cooperative constructive play. During this stage, the children take on the role of game designers, collaboratively crafting a play plan to achieve a shared objective. This method not only boosts creativity, but also cultivates a sense of responsibility and cooperation among the participants [25].
2. Cooperative constructive play. During this stage, the children become the focus, with the teacher adopting a supportive role. By stepping back, the teacher allows the children to team up and construct themes outdoors. This transition from direct instruction to facilitation involves the teacher observing, recording, and analyzing play dynamics to provide effective support. This observational stance helps the teacher recognize and nurture each child’s emerging skills and competencies, thereby optimizing the learning environment and interactions for both individuals and groups [26].
3. Drawing and sharing. This encourages the children to depict their play stories through drawing and group discussions. This crucial session enables children to articulate and refine their experiences, using drawings and discussions to deepen their understanding of their behaviors and interactions. This reflective practice solidifies the learning process and significantly enhances the children’s ability to effectively communicate their thoughts and emotions [27].

### 1.6. The Current Study

In China, the cultural emphasis on collective attention prioritizes the development of cooperative behaviors, particularly in educational settings. In 2021, the Ministry of Education of China introduced the initiative to “Revitalize Rural Education to Empower Rural Revitalization”. This policy underscores the importance of advancing preschool education accessibility and inclusivity, particularly in rural areas. Compared to urban regions, rural areas in China often face economic and educational disadvantages, with kindergartens in these areas typically suffering from less developed resources and a shortage of qualified teachers. Consequently, it is crucial to develop and implement educational programs tailored to the unique advantages of rural settings. These programs should be both practical and effective in their implementation to foster the growth of children and the development of kindergartens in these communities. Therefore, researching cooperative behavior in rural Chinese kindergartens is not only valuable but also essential in understanding and enhancing the educational impact in these underserved areas.

This study aims to evaluate the effectiveness and sustainability of cooperative constructive play designed on the Anji Play teaching model for improving cooperation among children aged 5–6 years. Our research questions are as follows:

RQ1: Is cooperative constructive play based on the Anji Play model effective in improving children’s cooperation?

RQ2: Can the cooperative strategies learned during the intervention be transferred to other play scenarios, such as role-playing games?

RQ3: Does the intervention based on Anji Play have a maintained effect on children’s cooperation?

The study employed a quasi-experimental design with an experimental group engaged in a 12-week intervention and a control group receiving routine teaching activities without any interventions. We assessed participants’ cooperation levels in constructive play at four time points: pre-, mid-, post-, and follow-up tests. Additionally, we measured the cooperative level and strategies pre-test and post-test in role-playing games. We hypothesized as follows:

**H1.**  *The intervention will significantly improve the experimental group children’s cooperation in constructive play.*

**H2.**  *The intervention will significantly enhance the experimental group children’s cooperation in role-playing games.*

**H3.**  *The effects of the intervention on cooperation will be maintained in the follow-up assessments.*

## 2. Materials and Methods

### 2.1. Participants

The study was conducted in a public kindergarten located in Hangzhou, Zhejiang Province, China. To determine the appropriate sample size, the researchers used a power analysis with the following parameters: a medium effect size (Cohen’s d = 0.5), two-sided testing, an alpha level (α) of 0.05, and a desired power (1 − β) of 0.80. The power analysis conducted using G*Power 3.1 indicated that a total sample size of 52 children would be required to achieve the desired power. To ensure robustness, the researchers decided to include a larger sample size. Therefore, two Daban classes, comprising a total of 60 children aged 5–6 years, were randomly assigned to either the experimental group or the control group, with each group consisting of 30 children. Specifically, the experimental group included 30 children (14 girls) aged 61–72 months (mean age = 66.27 months, SD = 3.53 months), and the control group included 30 children (15 girls) aged 61–73 months (mean age = 67.57 months, SD = 3.73 months).

Before the intervention, all children were screened to ensure that they were healthy and demonstrated typical developmental milestones. None of the children had previously participated in similar experimental interventions, ensuring baseline comparability.

An independent samples *t*-test confirmed no significant differences in age (*t* = −1.39. *p* > 0.05). Table 1 gives the descriptive results of the two groups on cooperation, cooperation level, and cooperation strategies before the intervention. We conducted an independent samples *t*-test to explore the differences between the groups for each outcome measure. Our analyses showed that there were no statistically significant differences between the experimental and control groups in terms of cooperation scores, the four dimensions of cooperation level, and the three dimensions of cooperation strategies, *p* > 0.05. The results showed that there were no significant differences between the groups, indicating homogeneity at the outset of the study.

Additionally, the educational background and experience of the teachers involved in both groups were similar, negating potential biases related to instructional quality. Both groups were led by classroom teachers holding a bachelor’s degree in preschool education, with six and eight years of teaching experience for the experimental and control groups, respectively. The teaching assistants in the experimental group had four years of experience, while those in the control group had three years. This similarity ensures that differences in child outcomes can more confidently be attributed to the intervention rather than to disparities in teacher qualifications or experience.

### 2.2. Development of Intervention

**Figure 1:**
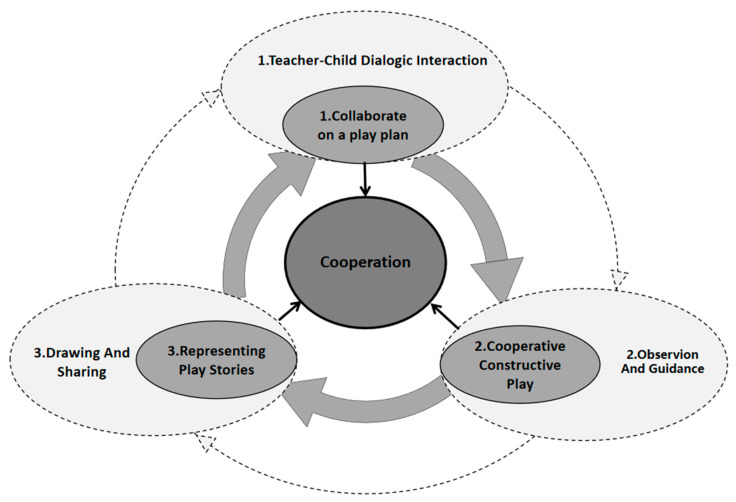
Framework of cooperative constructive play.

Drawing on the large-scale outdoor constructive play teaching model of Anji Play, we designed an intervention termed cooperative constructive play (CCP) to enhance children’s cooperative abilities by integrating the development of their cooperation levels and strategies into regular kindergarten play. This intervention embeds structured play activities within a flexible curriculum tailored to foster social interaction and collaborative problem-solving among children. The framework for CCP is depicted in Figure 1.

Figure 1 illustrates the dual-layer structure of CCP. The inner layer, delineated by dark grey solid lines, represents the children’s play zone. Here, children collaboratively plan their play activities, constructively engage during the play, and, afterward, articulate the narratives of their experiences. The outer layer, marked by light grey dotted lines, signifies the teachers’ support zone. Teachers facilitate the children’s play planning with guided discussions, observe and mentor the development of cooperation strategies during the activities, and help children articulate their play experiences through drawing and sharing sessions post-play. Each thematic unit under this model consists of 3–4 progressive cycles, with activities designed to build upon the experiences of the previous cycles, thereby enhancing the continuity and depth of learning.

**Figure 2:**
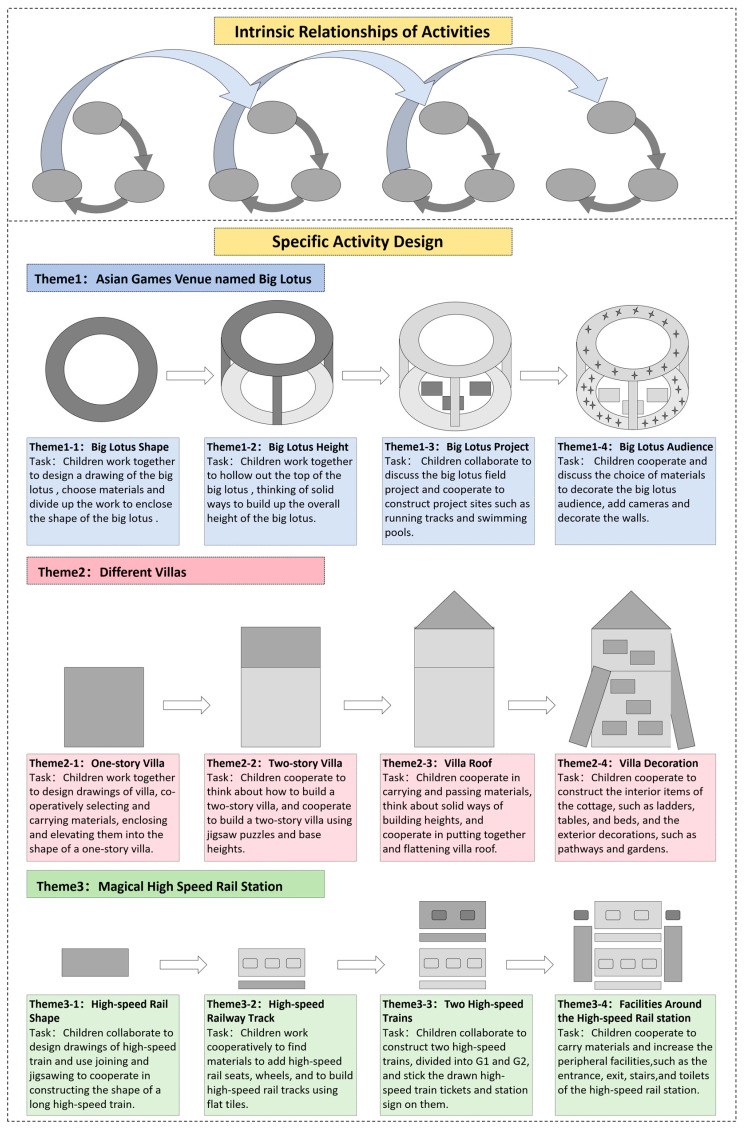
Illustration of the intrinsic relationships between activities and their specific designs.

The CCP program spans 12 weeks and is structured around three themes chosen to resonate with the children’s interests: the Asian Games Venues named Big Lotus, Different Villas, and Magical High-Speed Railway Station. These themes serve as the backdrop for the outdoor construction activities, with each theme being explored over four weeks. Every week introduces a mini-theme that adapts based on the dynamic interplay of the children’s ongoing constructions and interactions, supported by the teacher to facilitate progression toward completing the overarching theme. Figure 2 provides an illustration of the intrinsic relationships between activities and their specific designs.

Teachers integrate cooperation strategies into the educational objectives, ensuring they are embedded in every activity. By tailoring these strategies to the construction plans and the children’s spontaneous actions, as well as their prosocial and general strategies, are promoted while the use of mandatory strategies is minimized. Each session aims to foster not only physical and cognitive development but also social and cooperative skills. Teachers primarily use the following strategies:

First, teachers use heuristic dialogue to introduce cooperation strategies. Each play theme is generated based on the children’s interests and needs. During teacher–child dialogue interactions, teachers encourage children to think about construction, introducing cooperation strategies through discussion. For example, children learn to cooperate by observing and assisting peers who are moving long Anji boards. During cooperative constructive play, the learned strategies are applied, with teachers intervening as needed to guide the children in using the cooperative model.

Second, teachers document and reflect on the children’s cooperative behaviors to reinforce these strategies. They document behaviors through photos and videos, particularly noting spontaneous cooperation strategies. In the representation of play stories, children draw the cooperation strategies they used and share these with teachers and peers, using modeling methods to encourage others to adopt similar strategies. Photos and videos of cooperative strategies or conflicts are displayed on the interactive whiteboard to reinforce cooperation and introduce spontaneous strategies, thus reducing conflicts.

Third, teachers use cyclical reinforcement of cooperative strategies. Before each new constructive play, a teacher–child dialogue interaction session reinforces previously used cooperation strategies and introduces new ones. This cyclical reinforcement ensures that children experience and learn cooperative strategies before, during, and after play, enhancing their ability to work together with peers in construction activities.

The selection of materials each week is adaptive, based on the children’s interests and needs, incorporating ecological and flexible materials like Anji ladders, longboards, bricks, cups, and card stock. These materials are chosen to encourage creativity and ecological awareness among children, reflecting the program’s commitment to sustainable and child-centered learning environments. Please refer to Table S1 in the Supplementary Materials for details of the program.

The CCP intervention is held twice a week during the kindergarten’s designated outdoor playtime, with each intervention lasting between 60 and 80 min. To optimize engagement and outcomes, the children are divided into two groups of 15 each. The activities are structured in three stages: (1) Pre-play: Children engage in dialogue with teachers to collaboratively sketch their construction plans on white paper, outlining the shapes, the materials needed, and the roles for the day’s activities. (2) In-play: Children work together to bring their plans to fruition, with teachers stepping back to provide observational support and guidance as needed. (3) Post-play: After the constructive play, children draw and narrate their play stories, representing the cooperative challenges and strategies encountered. This session is aimed at reinforcing prosocial behaviors, encouraging strategic thinking, and preparing for future collaborative tasks. For a detailed breakdown of these stages, refer to Table 2.

### 2.3. Procedure

#### 2.3.1. Ethical Clearance

The first author’s university reviewed and approved the study. Invitation letters were sent to the participating kindergartens, and consent was obtained from the directors and classroom teachers. We notified all parents of the study, and they consented to their children’s participation. All parents and kindergarten teachers participating in the study signed a written consent form. The children in the participating kindergartens gave verbal consent to participate in the study, knowing that they could withdraw at any time.

#### 2.3.2. Six Stages

This study was conducted during the first semester of the 2023–2024 school year. The study was divided into six phases: pre-experiment, pre-test, intervention, mid-test, post-test, and follow-up test. A pre-experiment was conducted one week before the formal experiment. The purpose was to help the experimenters familiarize themselves with the testing process and adjust the intervention plan accordingly, based on any problems encountered during the pre-experiment. Ten children aged 5–6 years, including five boys and five girls, completed the pre-experiment procedure. Based on their performance and feedback, we selected the final intervention materials and program for the experiment. The 10 preschoolers who participated in the pre-experiment would not participate in the formal experiment.

A pre-test using the truck racing task was administered before the start of the experiment; in addition, the children’s behaviors in role-playing games were recorded. During the intervention phase, the experimental group was exposed to a 12-week cooperative constructive play intervention based on the Anji Play teaching model, while the control group was not exposed to any other interventions. The truck racing task was used in the mid-test, post-test, and follow-up test to measure the children’s cooperation. The children’s behaviors in role-playing games were recorded in the pre- and post-test stages.

We trained two assistant teachers, who were unaware of the purpose of the experiment, to test the children’s cooperation. To maintain ecological validity and prevent practice effects, we chose toy trucks of different colors but the same shape and size for each of the four tests. The children were randomly grouped into pairs, each choosing a color, and each started at the corresponding starting point and drove the truck simultaneously toward the other end. To control for extraneous variables, the experimental setting was an empty classroom with no remaining distractions, and the same graduate student was responsible for randomly grouping the children into pairs, with two children driving the toy trucks facing each other at each end of the three paths; the testers, unaware of the participants’ group assignments, administered all of the tests; and the test instructions were always explained by the main tester.

In addition, the children’s cooperation levels and strategies in role-playing games were measured using video observations conducted over two weeks, three times per week, for 10 min per child.

### 2.4. Measures

#### 2.4.1. Truck Racing Task

To assess children’s cooperation, we employed a classic experimental paradigm known as “truck racing” [28]. This task, adapted by Chinese scholars Lu and Huo [29] to study preschool children’s cooperative behaviors, is recognized as an effective tool for measuring cooperation in Chinese contexts. For this study, we created a simulated truck racing map and provided two toy trucks differing in color, shape, and size to facilitate the game. The track comprises three routes, two curved roads and a central straight road, each designed to be narrow enough to allow only one vehicle to pass at a time (see Figure S1).

The curved road is longer than the straight road, so taking the straight road is faster. According to the rules, only one car can be on the road at a time. The quickest and most cooperative method for both to reach their destination is for one person to wait while the other person drives the car through the straight road, and then the first person goes through it afterward. This requires mutual coordination and excellent cooperation, earning them 3 points for successfully cooperating. If, after discussion, one person takes the straight road to arrive quickly while the other makes a concession and takes the slower curved road, they score 2 points for successful cooperation. If both take the curved road and arrive slowly at the finish line to avoid collision, they score 1 point for successful collaboration. If both try to take the shortest route down the middle without communicating, resulting in a collision and the inability to pass, they score 0 points for failing to cooperate.

#### 2.4.2. Role-Playing Games Observation Tool

We drew upon the frameworks established in previous studies to assess cooperation levels and strategies during role-playing games. The dimensions of the cooperation levels were adapted from Cao’s research on the development of cooperation abilities in children’s role-playing games [30]. The dimensions of cooperation strategies were based on Yu’s observations and analyses of peer cooperation behaviors and strategies used by children aged 3–6 years in various daily activities [31].

Regarding the “Coding Manual for Cooperation Level and Strategies in Children’s Role-playing Games”, the rater used the “Record Sheet of Observations of Children’s Cooperation Level and Strategies” (see Tables S2 and S3 for details) to code children’s recorded behaviors. During each 10-min video of the role-playing games, instances of cooperative behavior were coded and scored. One point was awarded for each observed occurrence of cooperation at any level and an additional point was awarded for each observed use of a cooperation strategy.

Two assistant teachers were trained, and each video clip was evaluated by two raters, who evaluated the scores of each child’s level and strategies of cooperation according to the observation instructions, and finally averaged the scores of the two raters as the final scores.

### 2.5. Statistical Analysis

Statistical analyses were conducted using SPSS version 22 (IBM Corp., Armonk, NY, USA). Initially, descriptive statistics were calculated to summarize the cooperation scores. To examine the differences between the experimental and control groups, independent sample *t*-tests were employed. We assessed the effect of the cooperative constructive play (CCP) intervention by comparing the pre-test and post-test cooperation scores of the experimental and control groups using repeated measures ANOVA. The transfer effect of the intervention was analyzed by comparing the changes in cooperation levels and strategies in role-playing games from pre-test to post-test for both groups, using repeated measures ANOVA. To evaluate the maintenance effect of the intervention, we compared the follow-up cooperation scores of the experimental group with its post-test and pre-test scores.

## 3. Results

### 3.1. Testing of Hypothesis 1

To examine the intervention effects, a 2 (group: experimental, control) × 4 (time: pre-test, mid-test, post-test, follow-up test) repeated measures ANOVA was performed. There was a significant main effect of time on cooperation, [*F* (3, 174) = 34.05, *p* < 0.001, *η_p_*^2^ = 0.37]; a significant main effect of group on cooperation, [*F* (1, 58) = 14.47, *p* < 0.001, *η_p_*^2^ = 0.20]; and a significant interaction between time and group, [*F* (3, 174) = 16.33, *p* < 0.001, *η_p_*^2^ = 0.22].

A simple effects test for time revealed that in the experimental group, post-test cooperation scores were significantly higher than pre-test scores, as depicted in Table 3. Conversely, in the control group, there was no statistically significant difference between the post-test and pre-test scores. These findings affirm Hypothesis 1, indicating a significant training effect of the intervention.

### 3.2. Testing of Hypothesis 2

To evaluate Hypothesis 2, a 2 (group: experimental, control) × 2 (time: pre-test, post-test) repeated measures ANOVA was conducted focusing on cooperation levels and strategies. Significant interactions were observed for spontaneous, adaptive, and organized cooperation types, except for intentional cooperation, which showed no significant interaction (Table 4).

Further analysis through a simple effects test for time, detailed in Table 5, indicated that significant improvements were noted in the experimental group post-test compared to the pre-test for adaptive and organized cooperation. However, spontaneous cooperation showed a significant decrease. In the control group, changes over time were not statistically significant.

Moreover, interactions between time and group were significant for mandatory, general, and prosocial strategies (Table 4). A simple effects test showed that post-test scores for general and prosocial strategies were significantly higher than pre-test scores in the experimental group, and scores for mandatory strategies significantly decreased. In contrast, in the control group, only mandatory strategies showed a significant decline from pre-test to post-test; no significant changes were observed for general and prosocial strategies (Table 5). These results support Hypothesis 2, suggesting that the intervention effectively enhances children’s cooperation in role-playing games.

### 3.3. Testing of Hypothesis 3

To assess the maintenance effect of the CCP intervention, we compared the follow-up test scores of the experimental group with their pre-test and post-test scores (Table 3). The results indicated that scores at follow-up were significantly higher than both pre-test and post-test scores. This evidence supports Hypothesis 3, demonstrating a significant long-term maintenance effect of the intervention.

## 4. Discussion

This pioneering study evaluates the impact of cooperative constructive play (CCP) based on the Anji Play teaching model, significantly contributing to our understanding of how CCP aids cooperation among young children.

### 4.1. Impact of CCP on Children’s Cooperation

Our results demonstrate that CCP enhances cooperation in structured contexts and transfers effectively to other settings, such as role-playing games, with enduring effects. These findings align with Garaigordobil’s study [32], which also showed significant enhancements in prosocial behaviors and leadership among children engaged in similar cooperative play activities. Further validation comes from a study in China that reported notable improvements in cooperative behaviors among preschool children following cooperative game training [8]. This evidence collectively underscores the effectiveness of play-based interventions in fostering social skills among young learners.

In contrast to the cooperation intervention research conducted in China [7], which focused on younger children using theme-based joint block play in a teacher-led format, our study involves older preschoolers in a more child-centered approach under the Anji Play teaching model. Our intervention includes stages like teacher–child dialogic planning and cooperative construction that culminate in the creation of play stories using large outdoor Anji blocks, thus emphasizing a more immersive and socially interactive learning environment.

The theoretical underpinnings of our findings resonate deeply with Vygotsky’s concept of the zone of proximal development (ZPD) [33]. Vygotsky posited that children learn optimally in social settings, where they can model behaviors just beyond their current abilities with support from peers and teachers. The CCP environment created such a setting where children engaged in activities that not only challenged their current skills, but also encouraged the development of new cooperation strategies. This interaction not only fostered immediate cooperative skills but also enhanced higher psychological processes, such as problem-solving, perspective-taking, and metacognitive abilities. The scaffolding provided by peers and adults within CCP sessions exemplifies Vygotsky’s ZPD, facilitating the progression from assisted to independent performance in cooperative tasks.

The impact of CCP also aligns with Bandura’s social learning theory [34], which emphasizes the significance of observing and modeling behaviors within a cooperative context. In CCP sessions, children observed cooperative behaviors, such as sharing and negotiating, being modeled by their peers and teachers. This observational learning was reinforced through the shared success of their activities, leading to the internalization of cooperative behaviors. Bandura’s theory suggests that such modeled behaviors, when observed in a social context, are more likely to be adopted and reproduced by children, thereby enhancing their cooperative skills.

Moreover, the concept of transfer of learning is crucial in understanding the broader impact of CCP. Transfer of learning suggests that skills developed in one context can enhance performance in other areas [35]. This was observed as the children applied the cooperation strategies learned during structured CCP activities to more informal role-playing games, indicating a deep internalization of these skills that transcended the immediate learning environment. This transferability underscores the robustness of the cooperative skills developed through CCP.

According to Bronfenbrenner’s ecological systems theory [36], the setting of early childhood education plays a pivotal role in child development. Bronfenbrenner’s theory emphasizes the multiple layers of environment that influence a child’s development, from immediate settings like family and school (microsystem) to broader societal contexts (macrosystem). The kindergarten environment enriched by CCP served as a productive microsystem that significantly influenced children’s social interactions and overall development. By providing a structured yet flexible play environment, CCP facilitated interactions that supported both individual and collective growth, aligning with Bronfenbrenner’s emphasis on the dynamic interplay between children and their environments.

Overall, the integration of theoretical insights with our empirical findings underscores the effectiveness of constructive play interventions like CCP as potent educational tools. By strategically designing play activities that enhance cooperative interaction, educators can significantly influence the social and cognitive development of children, preparing them for more complex future interactions and collaborative tasks. This fusion of theory and practice not only confirms CCP’s effectiveness but also broadens our understanding of its applicability in diverse educational settings.

### 4.2. Educational Implications

The findings from this study demonstrate the efficacy and adaptability of the cooperative constructive play (CCP) program based on the Anji Play teaching model, offering valuable insights for early childhood educators and policymakers. Successfully integrated into regular kindergartens, the CCP program shows that its core elements can be maintained across diverse cultural and institutional contexts, suggesting that similar positive outcomes are widely achievable. This versatility underlines the potential for replication and broader implementation of the CCP, which could benefit a wider range of children.

Moreover, the role of educators in facilitating play proves critical. By transitioning from direct instructors to more observational and supportive roles, educators enable children’s freedom to explore and interact autonomously. This shift is crucial for nurturing sophisticated social competencies, and suggests that teacher training programs should focus on developing skills in facilitating play-based learning. These programs could include workshops on creating environments that encourage productive play and techniques for subtly guiding children.

Furthermore, the benefits observed underscore the need for policymakers to integrate play-based learning frameworks like Anji Play into standard preschool curricula. Similar success with Anji Play-based interventions is seen in enhancing other cognitive skills. For example, a study by Chen et al. found that the circling curriculum for metacognition training (CCMT), based on Anji Play, significantly improved the metacognitive abilities of 5- to 6-year-old children through a three-month intervention [24]. Supporting such initiatives could involve funding for resources, professional development for educators, and establishing mechanisms to monitor and evaluate the long-term impacts of these programs on child development. By embracing these insights, educators and policymakers can significantly enhance the effectiveness of early childhood education, ensuring that children develop the necessary skills for future success.

### 4.3. Limitations and Future Directions

While the findings of this study are promising, they come with limitations that affect their generalizability and point to further research needs. First, the sample size was relatively small and limited to a specific geographic region in China. This may affect the generalizability of our findings to other regions and cultural contexts. Future research should aim to include larger and more diverse samples to enhance the external validity of the results. The quasi-experimental design used limits the ability to establish causality. Future studies should utilize randomized controlled trials to enhance the reliability of the results. Employing a longitudinal model would also offer insights into the enduring impacts of cooperative play interventions on child development.

Furthermore, involving families in cooperative play interventions could improve outcomes, as parental engagement is crucial for reinforcing skills developed at school [37,38]. Additionally, comparing the CCP with other educational interventions could shed light on the most effective elements for fostering cooperation and social skills. There is also a need for more research on the specific mechanisms through which Anji Play influences children’s development. While our findings support the effectiveness of child-led, self-directed play, further studies should investigate the underlying processes that contribute to these outcomes. This could include examining the role of teacher facilitation, peer interactions, and the specific types of play materials used. By addressing these limitations and pursuing the suggested avenues for future research, we can deepen our understanding of play-based educational approaches and their potential to foster holistic child development.

## 5. Conclusions

This study provides valuable insights into the cooperation development of children aged 5–6, demonstrating the effectiveness of a three-month Cooperative Constructive Play (CCP) program based on the Anji Play teaching model. The program enhances cooperative skills in regular kindergartens, proving its adaptability and broad applicability. Through teacher–child interactions, free play, and sharing activities, the CCP not only promotes children’s cooperation but also equips educators with tools to support early social competencies. This makes the CCP a valuable model for fostering essential cooperative behaviors in young learners.

## Figures and Tables

**Table 1:** Homogeneity test before the intervention.

	Experimental Group (*n* = 30)		Control Group (*n* = 30)			
	M	SD	M	SD	*t*	*p*
Cooperation scores	1.30	0.54	1.27	0.87	0.18	0.86
Cooperation level						
Intentional	1.03	0.18	1.07	0.25	−0.58	0.56
Spontaneous	1.23	0.43	1.17	0.46	0.58	0.57
Adaptive	0.60	0.50	0.63	0.49	−0.26	0.80
Prosocial	0.37	0.49	0.30	0.47	0.54	0.59
Cooperation strategies						
Mandatory	0.53	0.78	0.47	0.78	0.33	0.74
General	0.93	0.91	0.90	0.71	0.16	0.88
Prosocial	0.63	0.62	0.70	0.60	−0.43	0.67

**Table 2:** Description of the three stages of the CCP intervention.

Stage	Teaching Goal	Teaching Content	Duration
Pre-play	Preparation for Cooperation Experience	Through reflective dialogue between teachers and children, the children are guided to initially discuss the co-construction program and try to collaboratively draw the constructed work.	10–20 min
In-play	Cultivating Cooperation Strategies	The teacher provides building materials, observes and records, and guides children to work together.	40 min
Post-play	Reflecting on Cooperation Behavior	The teacher encourages children to recall and characterize the process of play and to reflect on how they used cooperation strategies to solve problems in play.	20 min

**Table 3:** Simple effects test on cooperation scores.

Group	I (Time)	J (Time)	Mean Difference (I–J)	Std. Error	*p* ^b^	95% Confidence Interval for Difference ^b^	
						Lower Bound	Upper Bound
Experimental Group							
	PRE	MID	−0.43	0.10	<0.001	−0.65	−0.22
	PRE	POST	−0.90	0.13	<0.001	−1.17	−0.63
	PRE	FU	−1.13	0.14	<0.001	−1.42	−0.84
	POST	FU	−0.23	0.10	0.032	−0.45	−0.02
Control Group							
	PRE	MID	−0.07	0.07	0.326	−0.20	0.07
	PRE	POST	−0.17	0.08	0.057	−0.34	0.01
	PRE	FU	−0.20	0.09	0.031	−0.38	−0.02
	POST	FU	−0.03	0.06	0.573	−0.15	0.09

Note: PRE = Pretest, MID = Middle test, POST = Post test, FU = Follow-up test. ^b^ The mean difference is significant at the 0.05 level.

**Table 4:** Mean scores and indicators of intervention effectiveness for cooperation levels and strategies.

		Pre-Test		Post-Test		Group and Time Interaction Indicators		
		M	SD	M	SD	*F*	*p*	*η_p_* ^2^
Cooperation level								
Intentional						0.28	0.599	0.01
	Experimental (*n* = 30)	1.03	0.18	0.63	0.49			
	Control (*n* = 30)	1.07	0.25	0.73	0.45			
Spontaneous						4.23	0.044	0.07
	Experimental (*n* = 30)	1.23	0.43	0.70	0.47			
	Control (*n* = 30)	1.17	0.46	1.03	0.72			
Adaptive						25.41	<0.001	0.31
	Experimental (*n* = 30)	0.60	0.50	1.70	0.88			
	Control (*n* = 30)	0.63	0.49	0.70	0.54			
Organized						37.38	<0.001	0.39
	Experimental (*n* = 30)	0.37	0.49	1.93	1.14			
	Control (*n* = 30)	0.30	0.47	0.40	0.50			
Cooperation Strategies								
Mandatory						4.16	0.046	0.07
	Experimental (*n* = 30)	0.53	0.78	0.10	0.31			
	Control (*n* = 30)	0.47	0.78	0.33	0.61			
General						18.32	<0.001	0.24
	Experimental (*n* = 30)	0.93	0.91	1.50	0.94			
	Control (*n* = 30)	0.90	0.71	0.73	0.69			
Prosocial						51.27	<0.001	0.47
	Experimental (*n* = 30)	0.63	0.62	2.87	1.33			
	Control (*n* = 30)	0.70	0.60	0.83	0.70			

**Table 5:** Simple effects test on cooperation levels and cooperation strategies.

	Group	I (Time)	J (Time)	Mean Difference (I–J)	Std. Error	*p* ^b^	95% Confidence Interval for Difference ^b^	
							Lower Bound	Upper Bound
Cooperation level								
Spontaneous	Experimental (*n* = 30)	PRE	POST	0.53	0.12	<0.001	0.30	0.77
	Control (*n* = 30)	PRE	POST	0.13	0.16	0.403	−0.19	0.46
Adaptive	Experimental (*n* = 30)	PRE	POST	−1.10	0.18	<0.001	−1.47	−0.73
	Control (*n* = 30)	PRE	POST	−0.07	0.10	0.489	−0.26	0.13
Organized	Experimental (*n* = 30)	PRE	POST	−1.57	0.23	<0.001	−2.03	−1.10
	Control (*n* = 30)	PRE	POST	−0.10	0.07	0.184	−0.25	0.05
Cooperation strategies								
Mandatory	Experimental (*n* = 30)	PRE	POST	0.43	0.13	0.003	0.16	0.71
	Control (*n* = 30)	PRE	POST	0.13	0.06	0.043	0.00	0.26
General	Experimental (*n* = 30)	PRE	POST	−0.57	0.14	<0.001	−0.86	−0.28
	Control (*n* = 30)	PRE	POST	−0.17	0.10	0.096	−0.03	0.37
Prosocial	Experimental (*n* = 30)	PRE	POST	−2.23	0.28	<0.001	−2.80	−1.67
	Control (*n* = 30)	PRE	POST	−0.13	0.09	0.161	−0.32	0.06

Note: ^b^ The mean difference is significant at the 0.05 level.

## Data Availability

The data presented in this study are available on request from the corresponding author.

## Supplementary Materials

The following supporting information can be downloaded at: https://www.mdpi.com/article/10.3390/bs14070533/s1, Figure S1: road map of the trucking game; Table S1: main framework of the program of guided activities on cooperative constructive play; Table S2: coding manual for cooperation level and strategies in children’s role-playing games; Table S3: record sheet of observations of children’s cooperation level and strategy.
