# Supplementary material for: Enhancing Cooperation in 5–6-Year-Old Rural Chinese Children through Cooperative Constructive Play Based on Anji Play: A Quasi-Experimental Study
Source: Behav Sci (Basel). 2024 Jun 25;14(7):533. doi: 10.3390/bs14070533 (PMC11273849; doi:10.3390/bs14070533)

Table S1. Main Framework of the Program of Guided Activities on Cooperative Constructive Play

Play Themes	Week	Sub-theme	Training goal	Materials
Asian Games Venue named Big Lotus	1	Big Lotus Shape	Consciously cooperating, compensation	large-scale blocks
	2	Big Lotus Height	Consciously cooperating, demonstrating	large-scale blocks、cup
	3	Big Lotus Project	Reasoning, explaining, compromising	large-scale blocks、cup、card stock
	4	Big Lotus Audience	Taking turns, helping	large-scale blocks、cup、card stock、decorations
Different Villas	5	One-story Villa	Negotiating, discussing	short ladder、short board
	6	Two-story Villa	Suggesting,negotiating	long ladder、planks
	7	Villa Roof	Reminding,compensation	long ladder、short ladder、planks、short board
	8	Villa Decoration	Judging, and evaluating, taking turns	long ladder、short ladder、planks、short board
Magical High Speed Rail Station	9	High-speed Rail Shape	Negotiating,consciously cooperating	large-scale blocks
	10	High-speed Railway Track	Discussing， reminding	large-scale blocks、can
	11	Two High-speed Trains	Demonstrating 、 reasoning, explaining	large-scale blocks、can、decorations
	12	Facilities Around the High Speed Rail	Judging, and evaluating, suggesting	large-scale blocks、can、decorations、red flag

Table S2. Coding Manual for Cooperation Level and Strategies in Children's Role-playing Games

Evaluation dimensions	Operational definition
Cooperation level	
Intentional cooperation	There is an intention to cooperate but no specific act of cooperation. E.g., Xiaowei said to Xiaohong that you play the role of a waiter to serve the food, Xiaohong agreed but still fiddled with the cup in her hand without serving the food.
Spontaneous cooperation	Children play with verbal communication but the cooperation process is chaotic and susceptible to outside interference. E.g., Feifei was originally serving as a waiter helping the chef to pass the food, Lili called Feifei to come as a guest, and Feifei immediately went to be a guest.
Adaptive cooperation	The children play spontaneously and can cooperate to complete the basic goals of the game, but it is not stable and lasting. E.g., when Xingxing suddenly said she wanted Coke, not Sprite, Dingding immediately helped her to change a cup of Coke, and a moment later Xingxing said she was out of Coke, Dingding immediately helped her to fill it up.
Organized cooperation	There is a clear division of labor and cooperation in the children's play, and individuals can follow the organization's arrangements for action, interacting more and accomplishing common goals. For example, children plan together to assign roles, three children as guests, one as a waiter, and one as a chef, with a clear division of labor and strict adherence to roles until the end of the game.
Cooperation strategies	
Mandatory strategy	Commanding and threatening
General strategy	Discussing, suggesting, reminding, reasoning, explaining, compromising, obeying, judging, and evaluating
Prosocial strategy	Consciously cooperating, negotiating, demonstrating, helping, compensation, taking turns

Table S3. Record Sheet of Observations of Children's Cooperation Level and Strategies

Name of child： Date： Observer：

Time (min)	1	2	3	4	5	6	7	8	9	10	Total
Cooperation level											
Intentional cooperation											
Spontaneous cooperation											
Adaptive cooperation											
Organized cooperation											
Cooperation strategies											
Mandatory strategy											
General strategy											
Prosocial strategy											

Note. Time sampling is taken in this observation sheet and observations are made every 1 minute.

Figure S1. Road Map of the Trucking Game

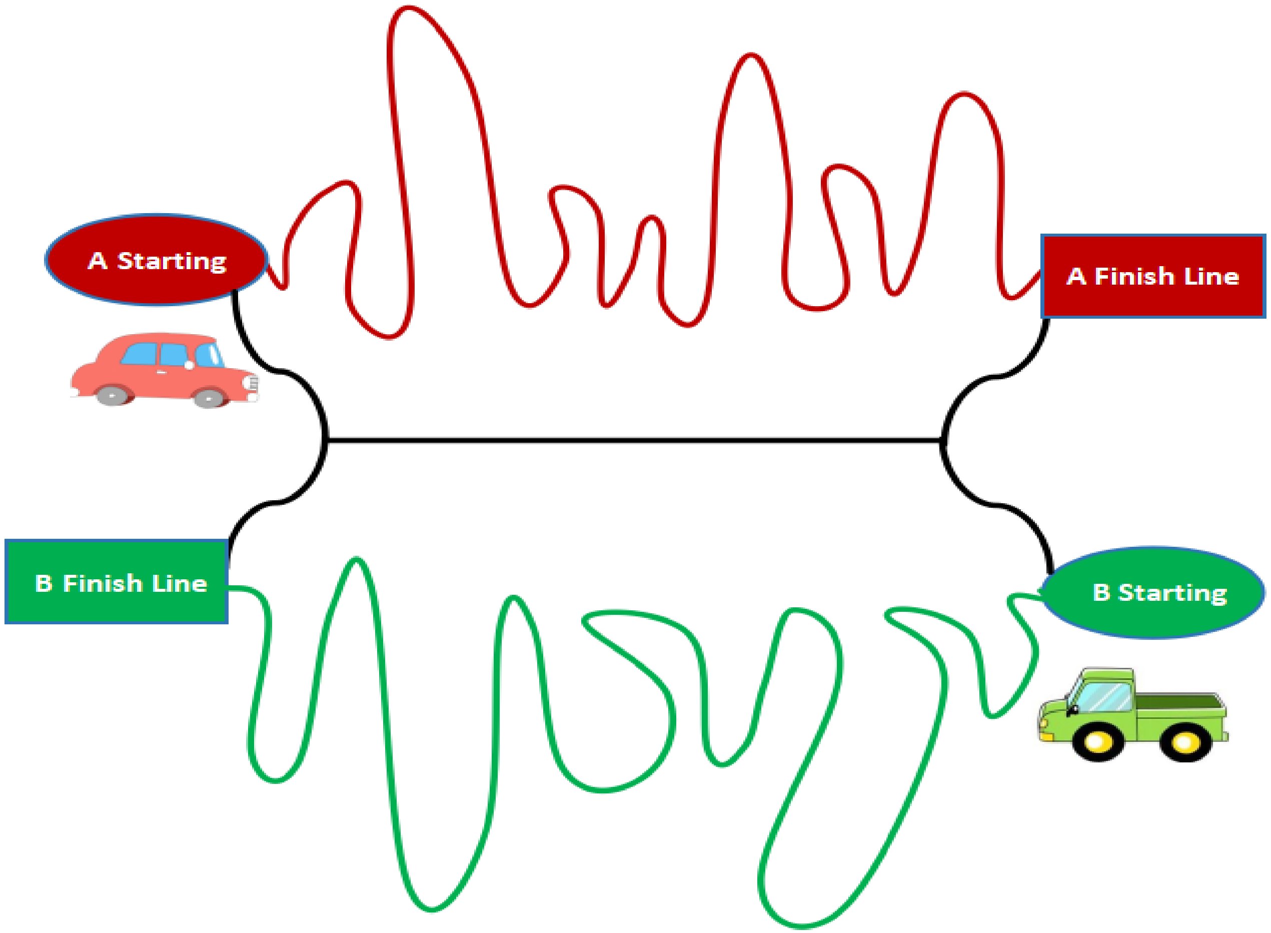

Supplement: Supplementary file 1 [file behavsci-14-00533-s001.zip › behavsci-3045864-supplementary.pdf]
